# Pan-disease blood protein profiles of rheumatic autoimmune diseases

**DOI:** 10.1038/s43856-026-01779-0

**Published:** 2026-07-13

**Authors:** Josefin Kenrick, Charlotta Preger, María Bueno Álvez, Maria Alejandra Ulloa, Göran Bergström, Antonella Notarnicola, Begum Horuluoglu, Angeles S. Galindo-Feria, Anna Smed-Sörensen, Anna Färnert, Anna Norrby-Teglund, Iva Gunnarsson, Marie Wahren-Herlenius, Marie Holmqvist, Leonid Padyukov, Karine Chemin, Lina Marcela Diaz-Gallo, Ingrid E. Lundberg, Elisabet Svenungsson, Vivianne Malmström, Lars Klareskog, Sofia Bergström, Mathias Uhlén, Peter Nilsson, Fredrik Edfors, Elisa Pin

**Affiliations:** 1 Department of Protein Science, SciLifeLab, KTH Royal Institute of Technology, Stockholm, Sweden; 2 Department of Molecular and Clinical Medicine, Institute of Medicine, Sahlgrenska Academy, University of Gothenburg, Gothenburg, Sweden; 3 Department of Clinical Physiology, Sahlgrenska University Hospital, Region Västra Götaland, Gothenburg, Sweden; 4 Division of Rheumatology, Department of Medicine Solna, Karolinska Institutet, Stockholm, Sweden; 5 Department of Gastroenterology, Dermatology and Rheumatology, Theme Inflammation and Aging, Karolinska University Hospital, Stockholm, Sweden; 6 Center for Molecular Medicine, Karolinska University Hospital, Stockholm, Sweden; 7 Division of Immunology and Respiratory Medicine, Department of Medicine Solna, Karolinska Institutet, Karolinska University Hospital, Stockholm, Sweden; 8 Department of Infectious Diseases, Karolinska University Hospital, Stockholm, Sweden; 9 Department of Medicine Huddinge, Center for Infectious Medicine, Karolinska Institutet, Stockholm, Sweden; 10 Department of Neuroscience, Karolinska Institutet, Stockholm, Sweden

**Keywords:** Proteomics, Rheumatic diseases

## Abstract

**Background:**

Systemic autoimmune rheumatic diseases (SARDs) are a heterogeneous group of autoimmune conditions characterized by immune system dysregulation leading to chronic inflammation and tissue damage. The overlapping clinical manifestations make differential diagnosis challenging, highlighting the need for novel biomarkers to facilitate early diagnosis, stratification, and personalized treatment.

**Methods:**

Five SARDs including idiopathic inflammatory myopathies (*n* = 210), rheumatoid arthritis (*n* = 84), systemic sclerosis (*n* = 100), Sjögren disease (*n* = 99), and systemic lupus erythematosus (*n* = 99), as well as healthy controls (*n* = 400) and controls with acute infectious diseases (*n* = 218) were selected for plasma protein profiling using Olink Explore 1536. Differential abundance analysis and machine learning were used to identify proteins with both known and novel association to SARDs.

**Results:**

The five SARDs share hundreds of proteins with consistently altered abundance compared to both healthy and infectious controls, reflecting common underlying molecular dysregulation. Despite the overlap, we identify multiple proteins with higher abundance specific to individual SARDs. Machine learning further enables accurate classification of the five SARDs, identifying a panel of 48 proteins with high discriminatory performance, several of which are also supported by differential abundance analysis.

**Conclusions:**

Altogether, this explorative cross-sectional study demonstrates the importance of a pan-disease approach, including also infectious and healthy controls, to identify robust and disease-informative protein panels for improved classification of SARDs. Protein levels from this study are available open access through the Human Protein Atlas, facilitating further plasma proteome research on autoimmune disease.

## Plain language summary

Systemic autoimmune rheumatic diseases (SARDs) are a group of diseases caused by a malfunctioning immune system that attacks and damages the body´s own tissues and organs. They can be difficult to distinguish and diagnose correctly because they share similar symptoms; therefore, additional molecular indicators could be helpful to aid in diagnosis. In this study, blood samples were collected from patients representing five SARDs. These samples were measured using a technology named Olink Explore, which measures around 1500 proteins in the blood at the same time. Using protein measurements from 592 patients, as well as from control groups, we identified proteins that may help distinguish the five diseases from one another. The proteins, if confirmed in future studies, could help clinicians in the diagnosis of these diseases with higher precision.

## Abstract (web summary)

Kenrick et al. investigate levels of more than 1500 proteins in blood across five systemic autoimmune rheumatic diseases using proximity extension assay. They demonstrate the importance of a pan-disease approach and identify disease-specific proteins that differentiate systemic autoimmune rheumatic diseases.

## Introduction

Systemic autoimmune rheumatic diseases (SARDs) are an umbrella term for several heterogeneous rheumatic disorders, all characterized by an immune system imbalance, which implies systemic organ and tissue involvement with overlapping clinical manifestations and serological features^1^. Despite advancements, the diagnosis of SARDs can be complicated and requires a multifaceted approach involving clinical evaluation from specialists alongside laboratory tests. SARDs include, among others, rheumatoid arthritis (RA), systemic lupus erythematosus (SLE), systemic sclerosis (SSc), Sjögren disease (SjD), and idiopathic inflammatory myopathies (IIM). Among these conditions, challenges in differential diagnosis stem from the wide range of clinical manifestations, involvement of multiple organs, and overlapping symptoms and signs such as fatigue, joint pain, and inflammation. The therapeutic opportunities are rapidly increasing for most of these diseases, with growing potential choice among different therapies for the various rheumatic diseases and their different subsets. There is thus a high need for additional biomarkers that can distinguish between these diseases and their subsets and contribute to the development of personalized, targeted therapies.

While autoantibodies are the hallmark of many SARDs and useful for diagnosis, they do not always reflect the ongoing inflammatory processes. Further, most currently used inflammatory markers are far too nonspecific to contribute to the differential diagnosis. Genetics remains useful for understanding disease pathogenesis, but is so far not useful in clinical diagnostics. Blood-based protein biomarkers provide a valuable complement to currently available tools for diagnosis and evaluation of disease activity, and they also offer insights into the often-unclear biological processes underlying the different diseases. New blood-based protein biomarkers may contribute both to molecularly driven differential diagnoses and to the identification of potential therapeutic targets.

Affinity proteomics has already been successfully applied to identify candidate biomarker panels for SLE, SSc^2–4^, and RA^5^. However, most of these studies focus on single diseases and comparisons with healthy controls, therefore not providing information on the overall disease specificity and usefulness for differential diagnosis of the candidate biomarkers. To ensure their reliability and clinical utility, plasma biomarkers should be evaluated in a pan-disease setting across diverse patient populations and pathological conditions to assess their specificity^6^. Within the SARD context, a recent study screened the level of 161 mainly immunoregulatory proteins in the blood of patients with SLE, ANCA-associated systemic vasculitis, RA, and SjD using antibody arrays, and reported a small number (*n* < 10) of disease-specific proteins that could differentiate the diseases^7^.

In the present study, we applied proximity extension assay (PEA) to screen 1472 proteins in the plasma of 592 patients with SARDs as part of the Human Disease Blood Atlas, namely SLE, RA, SjD, SSc, and IIM—as well as 400 healthy controls and 218 patient samples from three acute infectious diseases. This study provides a comprehensive analysis of the plasma proteome of five SARDs, and identifies single proteins and protein panels that can differentiate between different SARDs and from controls. Protein levels are available from this study in the open-access Human Protein Atlas (www.proteinatlas.org) as a resource for further research^6^.

## Methods

### Samples and patients

**Fig. 1:**
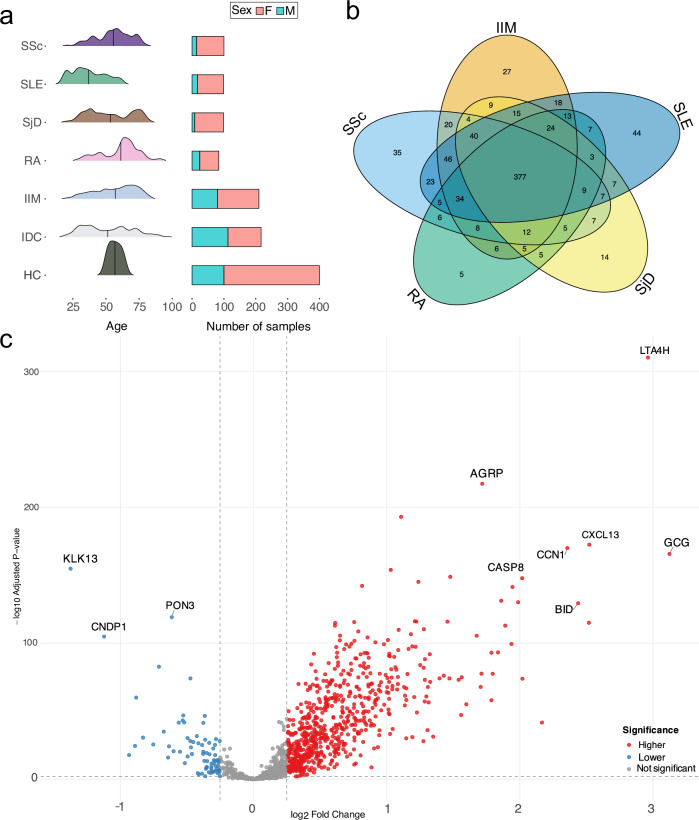
Cohort characteristics and autoimmune protein signatures. **a** Age and sex distribution for systemic sclerosis (SSc), systemic lupus erythematosus (SLE), Sjögren disease (SjD), rheumatoid arthritis (RA), idiopathic inflammatory myopathies (IIM), infectious disease controls (IDC), and healthy controls (HC). **b** Venn diagram showing the number of overlapping proteins from each individual SARD that had higher levels (adjusted *p*-value < 0.01, logFC > 0.25) compared to the healthy controls. **c** Volcano plot highlighting proteins with differential levels in the autoimmune cohort in comparison to healthy controls. Proteins with significantly higher levels (adjusted *p*-value < 0.01, logFC > 0.25) in the autoimmune cohort are shown in red, and lower levels (adjusted *p*-value < 0.01, logFC < −0.25) against healthy in blue.

The study included 592 patients with SARDs enrolled at the Rheumatology clinic of Karolinska University Hospital in Stockholm, Sweden and 400 healthy controls between ages 50–65 as part of the Swedish CArdioPulmonary bioImage Study (SCAPIS) study^8^ (Fig. 1a). To serve as an additional set of controls, 218 samples from patients with streptococcal soft tissue infection (*n* = 77), acute influenza virus infection (*n* = 98), and non-severe malaria^9^ (*n* = 43) were included, with the aim to account for non-autoimmune inflammation markers. The SARDs included in this study were IIM (*n* = 210), RA (*n* = 84), SjD (*n* = 99), SSc (*n* = 100), and SLE (*n* = 99) (Fig. 1a). Plasma EDTA was collected in K2 EDTA tubes, prepared through centrifugation at 2400×*g* for 7 min, and subsequently stored at –80 °C. Most samples were collected at the time of diagnosis and pre-treatment. Classification criteria for the diseases were based on the EULAR/ACR 2017 criteria for IIM^10^, the EULAR/ACR 1987^11^ or 2010^12^ criteria for RA, the EULAR/ACR 2016 criteria for SjD^13^, the EULAR/ACR 2013^14^ criteria for SSc, and the ACR 1982 revised classification and the 2012 SLICC criteria for SLE^15,16^.

Study participants were enrolled and samples collected according to the ethical principles outlined in the Declaration of Helsinki. All individuals gave written informed consent. Ethical permits were approved from the regional ethics committee in Stockholm, Sweden (protocol/Dnr 2005/792-31/4, 98-367, 2017/373-31/2, 2006/529/31, 2021-033005, 2015/1949-31/4, 2006/893-31/4, 2013/550-32, 2015/2200-32, 2016/1940/32, 2018/2354-32, 2019-03436, 2020-00859, 2020-04147, 2024- 00374-02), the ethics committee at Karolinska Institutet, Stockholm, Sweden (protocol/Dnr 03-556), the ethics committee in Lund, Sweden (no. 2021-00125), and the ethical review board of Göteborg, Sweden (proctocol/Dnr 407-15, 2015-06-25).

### Proximity extension assay

Protein levels were measured in plasma using the Olink Explore 1536 platform as part of the Human Disease Blood Atlas program^6^. Samples were fully randomized and normalized following the standard plate protocol defined by Olink Proteomics AB (Uppsala, Sweden), as described in Álvez et al. ^6^. Internal controls were used from the provider for intensity normalization and adjustment for inter-plate variation. The extension control was used for normalization of intensity signals, and protein levels are shown as normalized protein expression (NPX) units on a log2 scale. No further normalization was performed on the data after standard Olink NPX normalization. Samples were fully randomized and run on separate plates, and Olink’s inter-plate control was applied to adjust for plate-to-plate variation^17^. All samples were run in one batch. According to the recommendations from Olink Proteomics AB, LOD-based imputation was not considered, and the values below LOD were handled in the same manner as other values. Eight proteins were identified by quality control (QC) at Olink Proteomics AB as having a high-dose hook effect, and those were removed alongside 304 proteins that had a strong correlation (rho > 0.7) with them (Supplementary Fig. 1). The protein procollagen C-endopeptidase enhancer 1 (PCOLCE) was excluded due to assay QC warnings >50%, calculated by Olink QC software. Samples were removed if over 50% of NPX values had sample QC warnings, which occurs when internal controls from the provider deviate by ±0.3 NPX from the plate median. Of the 1158 proteins used in the final dataset after QC filtering, 476 proteins had no missing values, and 682 had at least one missing value, with the maximum percentage of missing values at 0.5% per sample, which was determined not to be disease-specific. *K*-nearest neighbor imputation was used for uniform manifold approximation and projection (UMAP) and machine learning in these instances.

### Statistics and reproducibility

Differential abundance analysis was performed using the *limma* (v3.62.2) package in R (v4.4.1), correcting for both age and sex^18^. Benjamini–Hochberg multiple hypothesis correction was used^19^. Each autoimmune disease was compared to a grouping of the other four autoimmune diseases, which served as a SARD control group. Each single SARD was also compared to both healthy controls and controls with infectious diseases, following the same differential abundance procedure. Adjusted *p*-values, as well as log2 of the fold change (logFC) for each protein and comparison, were calculated. Due to the large number of differentially abundant proteins within the comparisons, an adjusted *p*-value cut-off of 0.01 was chosen. A logFC cut-off of 0.25 was chosen to capture subtle patterns in the data, given the exploratory nature of the study.

Classification was performed using supervised multinomial GLMnet LASSO with *tidymodels* (v1.3.0) package. Nested cross-validation was used across five independent folds, split into training and testing sets for only SARD samples. Each training set contained an inner loop of 10 validation and training sets. Validation sets were used for tuning hyperparameters. NPX values were normalized, a near-zero variance filter was applied, and missing values were imputed within each training fold. Receiver operating characteristic (ROC) curves were computed for each fold, and the average area under the curve (AUC) was taken across the five folds. A confusion matrix was calculated based on the classification of the test set from each fold. The training set was used to determine the importance of individual features on the model using raw model coefficients and the *vip* (v0.4.1) package.

Gene set enrichment analysis was performed on the proteins with higher abundance compared to controls using *clusterProfiler* (v4.16.0) package in R. The background universe included the 1472 proteins left after QC on the Olink Explore 1536 dataset. All graphs were made using *ggplot2* (v3.5.1) in R. Uniform manifold approximation and projection (UMAP) was made using *uwot* (v0.2.3) package in R^20^.

Sample sizes are listed under “Samples and patients” subsection of the “Methods” section. Each sample has been tested once. Technical replicates were run as explained in “Proximity extension assay” subsection of the “Methods” section.

## Results

### Protein differential abundance analysis: SARDs vs. healthy controls and infectious disease controls

Initially, we analyzed all five SARDs versus healthy controls and infectious disease controls to identify plasma proteins that are unique to SARDs. In total, 377 proteins showed increased levels (adjusted *p*-value < 0.01, logFC > 0.25) in every autoimmune disease against healthy controls, with further overlap in the inter-SARD comparisons (Fig. 1b, Supplementary Fig. S2). Fewer proteins were found to have lower levels (adjusted *p*-value < 0.01, logFC < −0.25) than the healthy controls, with 16 proteins identified as shared across all SARDs (Supplementary Fig. S3).

In the overarching comparison of the grouped SARDs versus healthy controls, proteins involved in inflammatory response and response to oxygen-containing compounds had higher levels based on gene enrichment analysis (Supplementary Fig. S4). Among proteins that had higher levels in the grouped autoimmune diseases compared to healthy controls, we detected proteins involved in inflammatory and immune pathways, including leukotriene A4 hydrolase (LTA4H), cellular communication network factor 1 (CCN1), and C-X-C motif chemokine ligand 13 (CXCL13) (Fig. 1c).

When comparing the grouped autoimmune diseases with the infectious disease controls, gene set enrichment analysis revealed that the SARD group had higher levels of proteins involved in cell adhesion and morphogenesis, among other functions (Supplementary Fig. S5a and b). Among single proteins, cytokines and inflammatory proteins such as colony-stimulating factor 3 (CSF3) and interleukin 10 (IL-10) had lower levels in the autoimmune group in comparison to the infectious disease controls (Supplementary Fig. S5c).

### Protein differential abundance analysis: each SARD vs. other SARDs

**Fig. 2:**
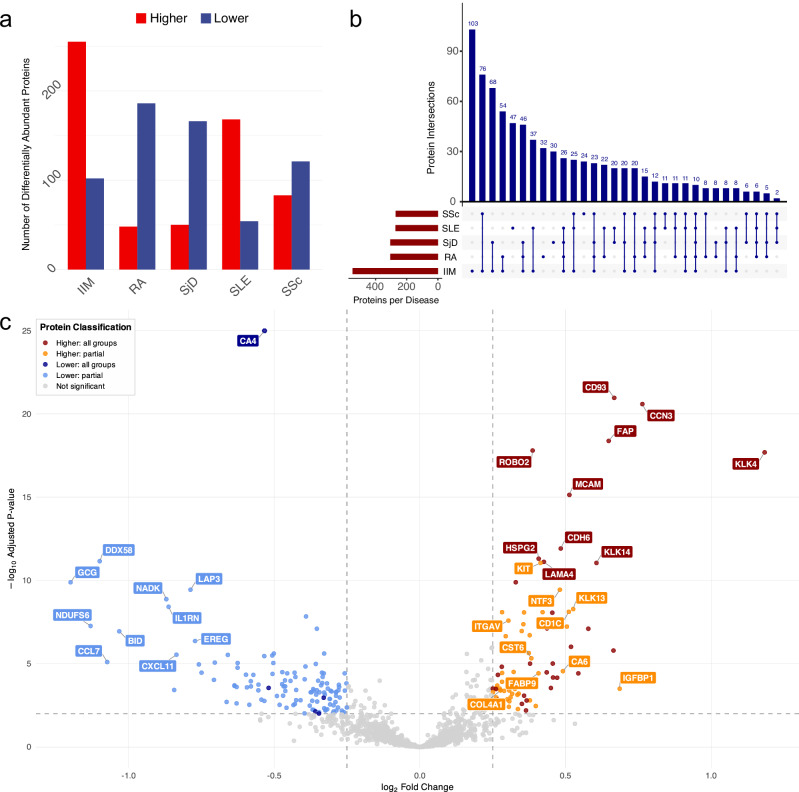
Differentially abundant plasma proteins across diseases. **a** Bar plot showing the number of proteins identified by differential analysis for each SARD using an adjusted *p*-value cutoff of 0.01, logFC> or <0, red = higher levels, blue = lower levels. **b** Upset plot showing proteins with higher abundance in each SARD based on cutoff-adjusted *p*-value < 0.01, logFC > 0.25. **c** Volcano plot for comparison of SSc to the grouped SARDs, annotated by comparisons to healthy and grouped infectious diseases. Dark red shows proteins significantly higher in SSc compared to all other groups (other autoimmune disease, healthy controls, and infectious disease controls), yellow shows proteins significantly higher in SSc than autoimmune either alone or in combination with one other group (healthy controls or infectious disease controls). Dark blue shows proteins significantly lower in SSc compared to all groups, and light blue shows proteins significantly lower in SSc compared to autoimmune, either alone or in combination with one other group (healthy controls or infectious disease controls).

The next stage of the analysis included differential analysis of each SARD against the other four SARDs. IIM was the disease with the highest absolute number of proteins with increased levels against all others, with RA having the highest absolute number of proteins with decreased levels (Fig. 2a, b, Table 1). Several proteins showed to be increased in level in more than one disease against other grouped SARDs, with the largest overlap between SSc and IIM (76 proteins) (Fig. 2b).

**Table 1:** Number of proteins significantly increased or decreased in single-SARD compared with other grouped SARDs (adjusted *p*-value < 0.01)

Disease	Increased levels	Decreased levels
	logFC > 0	logFC < 0
IIM	266	101
RA	23	138
SjD	33	134
SLE	135	44
SSc	85	127

To identify proteins specific for each single SARD, each elevated protein was annotated based on the result of the comparison against healthy and infectious disease controls (Fig. 2c, Supplementary Figs. S6–9). An example of a protein that may be disease-specific in SSc is kallikrein-4 (KLK4), which was elevated in comparison to the other SARDs as well as both control groups. In contrast, the protein mast/stem cell growth factor receptor, KIT, was elevated in SSc compared to the other SARDs and the grouped infectious diseases, but showed no significant difference relative to healthy controls. Finally, insulin-like growth factor binding protein 1 (IGFBP1) was higher in SSc than in other SARDs and healthy controls, but not compared to the infectious disease controls, suggesting it may be related to non-specific autoimmune inflammatory features (Fig. 2c). Similar patterns were found for all five SARDs (Supplementary Figs. S6–9). The results here will focus on proteins that were elevated in a specific autoimmune disease compared to all other SARDs.

**Fig. 3:**
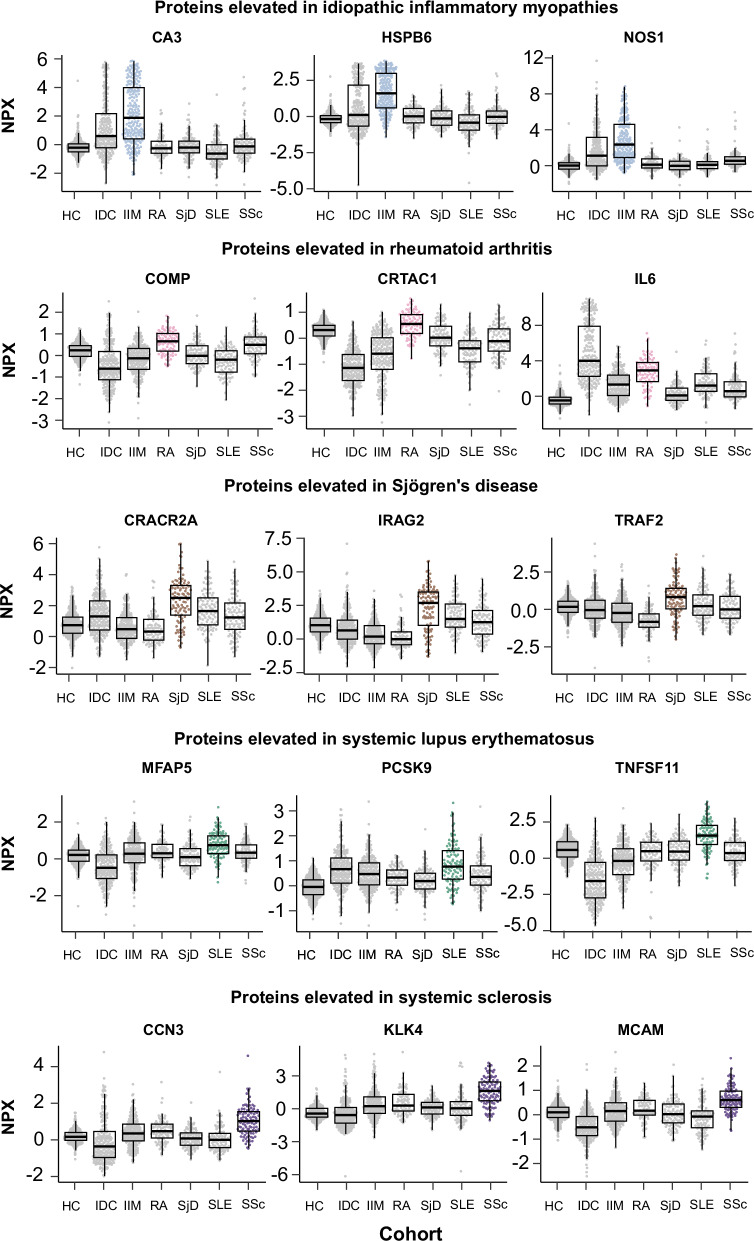
Plasma protein profiles across autoimmune diseases and controls. Each row represents one disease, with box plots showcasing a disease-specific pattern, having higher protein levels (normalized protein expression, NPX) than the other SARDs. Each dot represents one individual. IIM idiopathic inflammatory myopathies in blue with highlighted proteins carbonic anhydrase 3 (CA3), heat shock protein family B (small) member 6 (HSPB6), nitric oxide synthase 1 (NOS1); RA rheumatoid arthritis in pink with highlighted proteins cartilage oligomeric matrix protein (COMP), cartilage acidic protein 1 (CRTAC1), interleukin 6 (IL6); SjD Sjögren disease in brown with highlighted proteins calcium release activated channel regulator 2A (CRACR2A), inositol 1,4,5-triphosphate receptor associated 2 (IRAG2), TNF receptor associated factor 2 (TRAF2); SLE systemic lupus erythematous in green with highlighted proteins microfibril associated protein 5 (MFAP5), proprotein convertase subtilisin/Kexin type 9 (PCSK9), TNF superfamily member 11 (TNFSF11); and SSc, systemic sclerosis in purple with highlighted proteins cellular communication network factor 3 (CCN3), kallikrein related peptidase 4 (KLK4), melanoma cell adhesion molecule (MCAM). The gene name of selected proteins is visible in the plot.

In SLE, we identified higher abundance for immune-related proteins, such as microfibril-associated protein 5 (MFAP5) and proprotein convertase subtilisin/kexin type 9 (PCSK9) (Fig. 3, Supplementary Fig. S6). In addition, TNF superfamily member 11 (TNFSF11), also known as RANK ligand, was elevated in SLE compared to all other groups (Fig. 3). Among the proteins with increased abundance, SLE shared 11 with SSc (e.g., RBP2, FABP2) and 37 with IIM (e.g., VSIG4, IL-15).

Differential analysis for RA identified proteins involved in deep zone cartilage, such as cartilage acidic protein 1 (CRTAC1), and cartilage oligomeric matrix protein (COMP), despite having fewer differentially abundant proteins compared to the other SARDs (Fig. 3). Additionally, the established marker and therapeutic target interleukin 6 (IL6) was identified, despite being unsurprisingly higher in the infectious diseases^21^ (Fig. 3, Supplementary Fig. S7). Proteins identified from gene enrichment were involved in processes such as embryo development, integrin-mediated cell adhesion, and peptidyl serine phosphorylation (Supplementary Fig. S7).

In SjD, the transporter protein inositol 1,4,5-triphosphate receptor-associated 2 (IRAG2) had higher levels in comparison to the other SARDs. It has a connection to the immune system through its suggested role in the major histocompatibility complex (MHC), indicating a potential link to autoimmunity^22^ (Fig. 3, Supplementary Fig. S8). Other proteins found elevated in SjD were tumor necrosis factor ligand superfamily member 11 (TRAF2), a TNFR receptor, and EF-hand calcium-binding domain-containing protein 4B (CRACR2A), a calcium-binding protein.

As stated, IIM had numerous differentially abundant proteins, and notably, many of those with higher levels were specific to muscle tissue, such as nitric oxide synthase 1 (NOS1), carbonic anhydrase 3 (CA3), and heat shock protein family B (small) member 6 (HSPB6)^23^ (Fig. 3, Supplementary Fig. S9). Gene set enrichment revealed proteins involved in DNA damage response, cytokine-mediated signaling pathways, and macrophage activation, among others (Supplementary Fig. S9).

Previously mentioned proteins KLK4 and MCAM, found elevated in SSc, have roles in enamel formation and cell adhesion, respectively^24,25^. Lastly, cellular communication network factor 3 (CCN3), which impacts a variety of tissue-building functions, including cell proliferation, adhesion, migration, and differentiation, was found to be elevated in SSc compared to all controls (Fig. 3)^26^. Gene set enrichment identified biological processes of cell adhesion, cell junction assembly, and regulation of the notch signaling pathway (Supplementary Fig. S10).

### Machine learning classification

Machine learning multiclassification was applied with a nested cross-validation strategy, a supervised approach using a GLMnet LASSO regression model to classify all SARDs simultaneously. This helped identify complex patterns between the different diseases and allowed for the selection and ranking of protein features, thus complementing the differential abundance analysis.

**Fig. 4:**
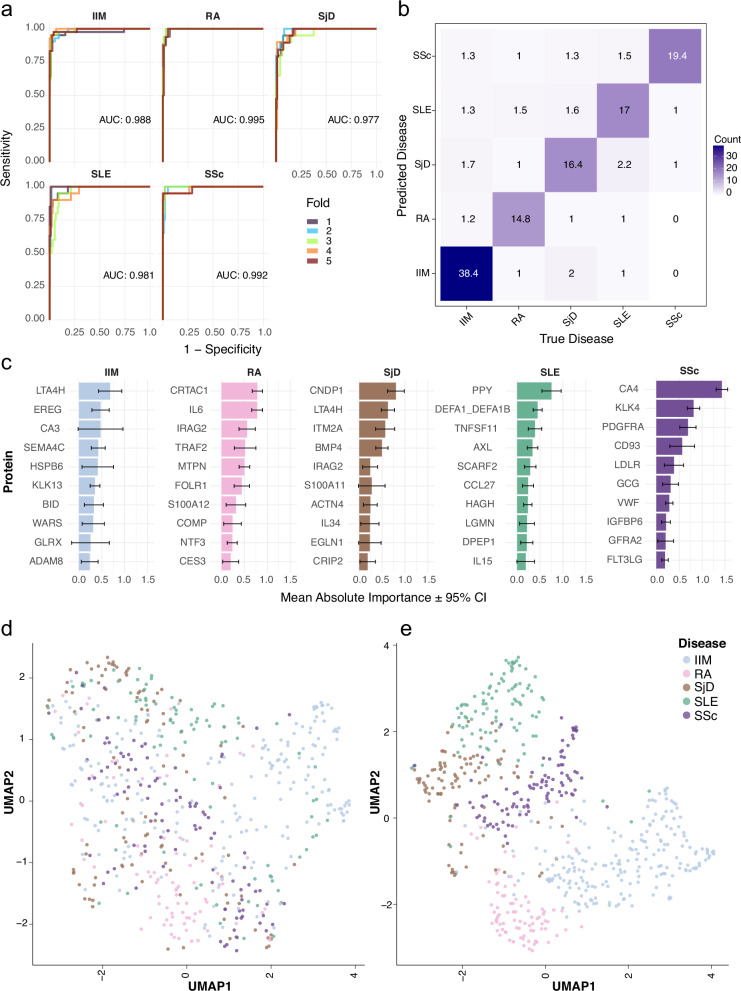
Pan-autoimmune classification using machine learning. **a** ROC curves of model performance across the five folds with the average AUC for each disease. **b** Confusion matrix across the folds with the average count of misclassified cases. **c** Mean absolute importance with 95% confidence interval of the top 10 important proteins by disease from the five folds. **d** UMAP dimensionality reduction plots shown with all proteins in the dataset and **e** after filtering for the top 10 features deemed important for each disease by machine learning (*n* = 48).

Across the five independent folds, ROC curves indicated high performance of the model, with average AUCs on the test set ranging from 0.98 to 0.99 (Fig. 4a). From the confusion matrix, the disease most often misclassified was SjD, still with only a few cases (Fig. 4b). From the training set, we identified the top features for classification of each disease (Fig. 4c). These top features, or important proteins, are identified via the absolute value of raw model coefficients ranging from 0 to 1.1643. Notably, protein leukotriene A4 hydrolase (LTA4H) was considered important for the classification of both IIM and SjD, while inositol 1,4,5-triphosphate receptor-associated 2 (IRAG2) appeared for SjD, SLE, and RA. These results confirm the higher level of IRAG2 found in SjD and SLE compared to the healthy and infectious disease controls in the differential abundance analysis (Fig. 2c). Similarly, LTA4H was identified in the differential abundance as having the highest levels when compared to HC for all SARDs. The performance of the classifier was high, with mostly predicted cases matching true cases. From the GLMnet results, the proteins included in the classifier were derived especially from SjD (*n* = 138), followed by IIM, SLE, SSc, and RA (Supplementary Data). Using the top 10 proteins deemed important for the classification of each disease, a total of 48 proteins were selected; the UMAP generated using the dataset of 48 proteins showed improved separation of disease clusters compared to using the whole dataset as input (Fig. 4c–e).

### Combining differential abundance and machine learning for disease-specific protein profiles

**Fig. 5:**
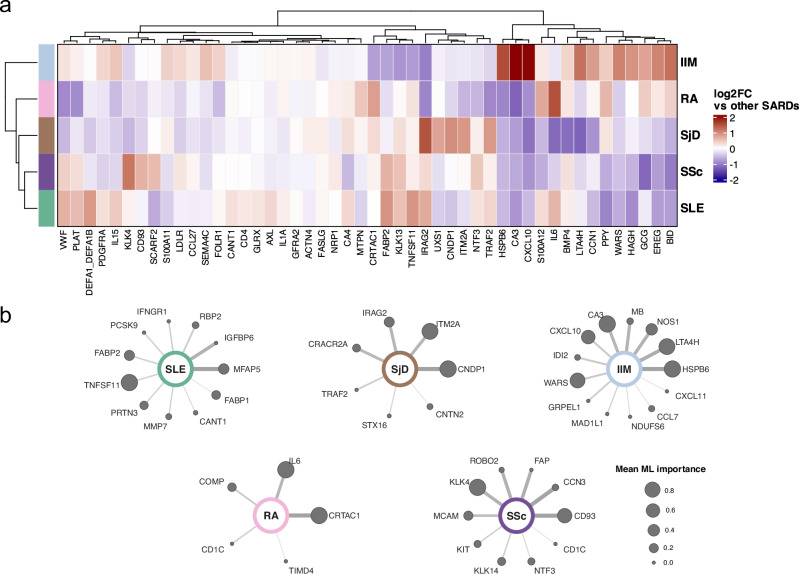
Disease-specific proteins were identified via both differential analysis and machine learning. **a** Heatmap of the logFC from differential abundance analysis of each disease against other SARDs for the 48 proteins identified as important in LASSO. **b** Network visualization of proteins identified by both methods associated with each disease, with line thickness corresponding to the significance of differential analysis and the protein dot size corresponding to the mean importance score.

A comparison of the results from the differential abundance and machine learning analysis was performed with the aim of selecting top-ranked proteins showing increased levels in one specific SARD compared to other SARDs and both healthy and infectious disease controls, therefore showing the highest level of specificity. Results showed that IIM had the highest overlap of proteins identified by both differential analysis and machine learning, followed by SLE and then SSc, RA, and SjD. Twelve proteins were identified as having higher levels than all controls based on differential abundance analysis and identified via machine learning in all five independent folds (Table 2). This corresponds to two to four proteins per SARD, with IRAG2 being selected in both SjD and SLE. Additionally, a heatmap of the top 48 important machine learning proteins showcases the logFC from the inter-SARD differential analysis, with selected proteins being highlighted for each disease in a network visualization (Fig. 5).

**Table 2:** Most relevant proteins elevated per disease selected by both differential abundance analysis and in each fold of machine learning

Disease	Gene name	Protein name	Uniprot ID	Disease vs. Healthy	Disease vs. Infectious disease	Machine learning mean importance
				logFC (adjusted *p*-value)	logFC (adjusted *p*-value)	
IIM	LTA4H	Leukotriene A-4 hydrolase	P09960	3.74 (3e−26)	0.89 (3e−11)	0.64 ± 0.38
	CA3^58^	Carbonic anhydrase 3	P07451	2.4 (3e−48)	0.77 (5e−05)	0.48 ± 0.41
	EREG	Proepiregulin	O14944	1.82 (9e−53)	0.60 (7e−05)	0.47 ± 0.31
RA	CRTAC1^59^	Cartilage acidic protein 1	Q9NQ79	0.20 (2e−3)	1.39 (1e−26)	0.82 ± 0.21
	COMP	Cartilage oligomeric matrix protein	P49747	0.37 (1e−07)	0.77 (4e−10)	0.23 ± 0.17
SjD	IRAG2^a^	Inositol 1,4,5-triphosphate receptor-associated 2	Q12912	1.44 (7e−12)	1.48 (1e8−11)	0.27 ± 0.17
SLE	AXL^30,31,60–62^	Tyrosine-protein kinase receptor UFO	P30530	0.75 (2e−21)	0.37 (8e−06)	0.36 ± 0.16
	TNFSF11	Tumor necrosis factor ligand superfamily member 11	O14788	0.66 (2e−4)	2.65 (2e−29)	0.33 ± 0.18
	IRAG2^a^	Inositol 1,4,5-triphosphate receptor-associated 2	Q12912	1.18 (4e−09)	0.66 (2e−3)	0.29 ± 0.12
SSc	KLK4^63^	Kallikrein-4	Q9Y5K2	2 (7e−34)	1.84 (7e−22)	0.89 ± 0.06
	CD93	Complement component C1q receptor	Q9NPY3	1.12 (1e−30)	0.98 (6e−20)	0.58 ± 0.28
	FABP2	Fatty acid-binding protein, intestinal	P12104	0.35 (5e−3)	1.3 (2e−10)	0.15 ± 0.12

^a^This protein was identified as important in more than one disease.

Altogether, our results provide novel insight into the plasma proteome of five SARDs, showing many proteins with different levels across autoimmune disorders, and with IIM and SLE having the highest number of proteins elevated in plasma when compared to other SARDs and controls. We demonstrate the relevance of using infectious disease controls in addition to healthy controls, particularly in the context of autoimmunity, when strong inflammatory responses might share characteristics with an acute infection. We showed that using the top proteins identified by machine learning allowed us to distinguish the SARDs from each other. Lastly, we present a short list of proteins selected both by differential abundance analysis and machine learning as the most specific proteins for every single disease.

## Discussion

This study leveraged the proximity extension assay (PEA) to analyze the plasma proteome in a large cohort of patients with five different SARDs in a pan-autoimmune disease setting. The results of differential abundance and machine learning analyses demonstrated that, despite having clinical and proteomic similarities, we were able to identify a subgroup of proteins specific to each of the SARDs.

There were many differentially abundant proteins, some with increased abundance and some with decreased abundance, in each disease compared to all other SARDs. In this study, we focused primarily on those with increased levels in each of the studied diseases, respectively, as these would be more easily translated into diagnostic biomarkers.

Systemic lupus erythematosus (SLE) and idiopathic inflammatory myopathies (IIM) showed the greatest number of proteins with elevated levels in the differential abundance analysis. In SLE, dysregulation of apoptosis and an insufficient clearance of intracellular proteins and nuclear debris have been reported to be involved in the pathogenesis of the disease^27^. Thus, this could be an explanation for the higher levels of proteins found in the plasma proteome of SLE patients. Regarding IIM, muscle fiber necrosis and regeneration are characteristic of most patients with inflammatory-active disease, which could result in the release of more proteins into the plasma.

Several members of the matrix metalloproteinase (MMP) family were identified in our study, with increased levels in SLE. The association between MMP and SLE has been suggested before^27,28^. In addition, the MMP-related protein MFAP5, which aids in fibroblast function and scar formation, was also identified in association with SLE through both methods^29^. Neutrophil defensin 1 (DEFA1_DEFA1B), an anti-microbial peptide found in the epithelia of mucosal surfaces, and tyrosine-protein kinase receptor UFO (AXL) both have known roles in SLE, and these were among the top proteins for SLE in machine learning and differential abundance^30–34^. Interestingly, both tumor necrosis factor receptor superfamily member 11A (TNFRSF11A aka RANK), and its ligand TNFSF11, part of the NF-κβ pathway and critical for bone density, were found to have significantly higher levels in SLE compared to the other autoimmune diseases^35^.

Several proteins in IIM had higher levels against the infectious disease controls (IDC), most notably those enriched in skeletal muscle tissue^23^. These proteins ranged from having roles in muscle function (HSPB6) to cholesterol synthesis (IDI2)^36,37^. Other key proteins identified include potential autoantigens, such as tryptophanyl-tRNA synthetase (TrpRS/WARS1). TrpRS is a member of the aminoacyl-tRNA synthetases, a family with several known autoantigens in IIM^38^. In addition, NADH:ubiquinone oxidoreductase subunit S6 (NDUFS6), a mitochondrial protein connected to complex I in the respiratory chain, was one of the proteins that showed a higher level in IIM in our study^39^. This is of interest in connection to a recent study from our group, which found autoantibodies against NDUFA11, another protein part of the same complex^40^.

In contrast, many of the higher-abundance proteins in IIM appeared in comparison to the other SARDs but had lower levels than the IDC, such as guanylate binding protein 2 (GBP2), involved in innate immunity, and chemokine ligands CXCL10 and CCL7^41^. These proteins involved in immunity and inflammation are higher in IIM than in other SARDs, possibly suggesting higher systemic inflammatory levels than other autoimmune diseases.

Several of the proteins with higher levels in systemic sclerosis (SSc) are involved in tissue synthesis, such as KLK4 of the kallikrein family and CCN3 involved in fibrosis. Other common categories included proteins expressed in endothelial cells, such as MCAM/CD146 and CD93^42–45^. The soluble version of CD93 has been shown to correlate with disease severity and activity of SSc, which we identified via both differential analysis and machine learning methods^46^. Fibroblast activation protein (FAP) showed high levels in SSc in our study, likely reflecting the involvement of fibroblasts in mechanisms of fibrosis and tissue repair underlying SSc^47^. However, FAP is better known by its role as a tumor marker^48^. The interplay between SSc and cancer is well established^49^ and evident in the results from the identification of multiple cancer-related proteins, including FAP as well as CDH6^50^, CD93^51^, CCN3^52^, MCAM^25^, and KLK4^53^.

For Sjögren disease (SjD), many of the proteins identified were novel in relation to the disease but already reported to be of relevance in cancer biology. Specifically, syntaxin 16 (STX16^54^), TNF receptor-associated factor 2 (TRAF2^55^), and calcium release-activated channel regulator 2A (CRACR2A^56^) have all been implicated in lymphoid cell and immune-related processes and were identified through both differential analysis and machine learning.

The machine learning generated high AUCs and a low level of misclassification between the diseases. Additionally, using the top-ranked proteins identified from the machine learning, we were able to visibly improve the separation between the different diseases in a high-dimensional space, suggesting the relevance of these proteins in differentiating between the SARDs.

Our study demonstrated the importance of using a pan-disease approach and inclusion of healthy and infectious disease controls to examine the proteome of SARDs in order to better understand the biology underlying these complex and oftentimes co-occurring diseases and identify disease-specific proteins as candidate markers. Furthermore, the combination of differential analysis and machine learning is a powerful technique to identify robust candidate markers.

The broad scope and ambitious aim of this study naturally lend to limitations. Firstly, the choice of the control group is crucial for identifying targets, and in this vein, it is important to validate our data using independent healthy and disease cohorts. Furthermore, the number of patients in each cohort is imbalanced, and therefore, machine learning analysis should be interpreted with caution. We utilized hyperparameter tuning and cross-validation to account for this, but there are still risks of overfitting.

Notably, all the rheumatic diseases investigated in this study can be divided into subsets that are different concerning clinical as well as molecular features. Future studies warrant analysis to address differences between serological and clinical subgroups, as well as disease status based on activity. In addition, future studies are warranted to investigate more in depth the differences between the diseases and the control groups with regard to clinical manifestations.

Despite the high sensitivity of the proximity extension assays, the broad dynamic range poses a challenge in the accurate measurement of proteins with extremely high or low concentrations in blood.

In the context of autoimmune diseases, there are often autoantibodies in the blood. These may interfere with the immunoassay by binding to the protein target of the autoantibody itself or binding to the antibody used for detection, such as in the case of rheumatoid factor^57^. This is worth investigating in a future study.

While in this study we focused on proteins with increased abundance in specific diseases, this decision could be limiting, as other proteins may hold valuable insights into the underlying biology of these autoimmune diseases.

As stated, these diseases are overlapping and share many clinical manifestations and are sometimes co-occurring. To get a better overall understanding of the disease mechanisms and pathogenesis and to move towards disease re-classification and more tailored treatment, it will be of interest in a future study to use a combination of clinical manifestations, autoantibody patterns, and protein biomarkers to identify novel molecularly defined disease subsets for future precision medicine efforts.

## Conclusions

Overall, our analysis confirmed the high similarity between the different systemic autoimmune rheumatic diseases on the protein level, which is not surprising given the frequent clinical overlap of some manifestations but also the common co-occurrence of some of these SARDs. Despite this, we identified individual plasma proteins that showed disease specificity, as well as protein combinations enabling discrimination among all five diseases. These protein profiles might aid in distinguishing unique biological mechanisms of these diseases. We demonstrated that a pan-disease perspective is critical for identifying SARD-specific biomarkers that are not solely due to inflammation. This data, also available open access through the Human Protein Atlas, represents a resource for further studies. In the future, elucidating differences between subtypes or clinical manifestations of disease is vital for further understanding the underlying biology of these SARDs.

## Supplementary information

Supplemental Information

Description of Additional Supplementary files

Supplementary Data 1-4

## Data Availability

Boxplots of the NPX values can be found open access on The Blood Resource of the Human Protein Atlas (https://www.proteinatlas.org/humanproteome/blood). Differential analysis and machine learning results are available in the supplementary material. Source data for all figures apart from Figs. 1a and 4 are available in the supplementary material. Source data for Fig. 3 is aggregated by disease, and NPX values are randomized. Source data for Figs. 1a and 4 cannot be shared publicly because they contain sensitive personal information, which is protected by GDPR. Data access can be made available for validation purposes upon reasonable request to the corresponding author.

## Code availability

All code for the data analysis and visualization is available on https://github.com/jjkenrick/pan-autoimmune-protein-profiling.git.
