# Supplementary material for: Pan-disease blood protein profiles of rheumatic autoimmune diseases
Source: Commun Med (Lond). 2026 Jul 13;6:390. doi: 10.1038/s43856-026-01779-0 (PMC13365237; doi:10.1038/s43856-026-01779-0)
Supplement: Supplementary file 1 — Supplemental Information [file 43856_2026_1779_MOESM1_ESM.pdf]

SUPPLEMENTARY INFORMATION

Pan-disease blood protein profiles of rheumatic autoimmune diseases

Josefin Kenrick, Charlotta Preger, María Bueno Álvez, Alejandra Ulloa, Göran Bergström, Antonella Notarnicola, Begum Horuluoglu, Angeles S Galindo-Feria, Anna Smed-Sörensen, Anna Färnert, Anna Norrby-Teglund, Iva Gunnarsson, Marie Wahren-Herlenius, Marie Holmqvist, Leonid Padyukov, Karine Chemin, Lina Marcela Diaz-Gallo, Ingrid E. Lundberg, Elisabet Svenungsson, Vivianne Malmström, Lars Klareskog, Sofia Bergström, Mathias Uhlén, Peter Nilsson, Fredrik Edfors, Elisa Pin

SUPPLEMENTARY FIGURES

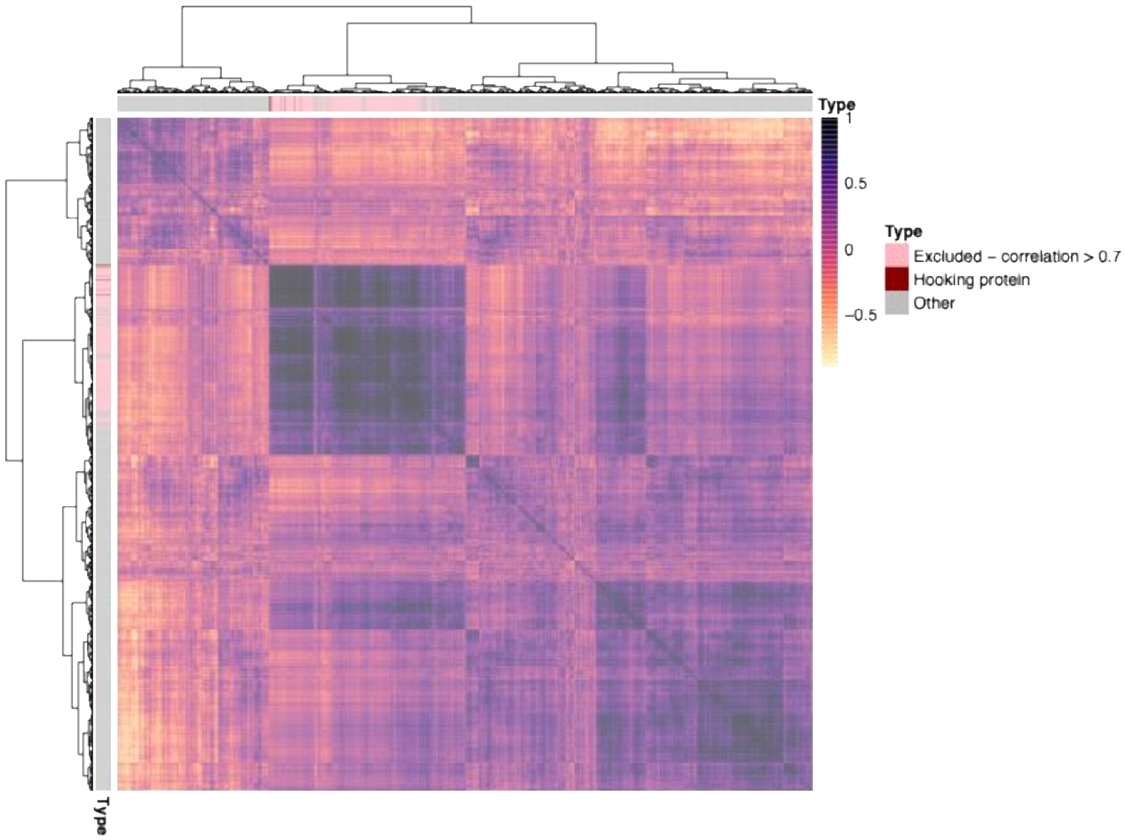

Supplementary Fig. S1*: Heatmap showing Pearson correlation of all proteins in the Olink Explore 1536 dataset, with annotations in dark red if a protein was identified as a hook protein or in pink if correlation with hook proteins was > 0.7. All other proteins annotated in grey were used in the final dataset. The eight hook proteins include: dual adaptor of phosphotyrosine and 3-phosphoinositides 1 (DAPP1), Mesencephalic astrocyte derived neurotrophic factor (MANF), Protein tyrosine phosphatase non-receptor type 6 (PTPN6), Phosphomevalonate kinase (PMVK), Phosphoribosyl transferase domain containing 1(PRTFDC1), Tubulin folding cofactor B (TBCB), Sulfotransferase family 1A member 1 (SULT1A1), and Cyclin dependent kinase inhibitor 2D (CDKN2D).*

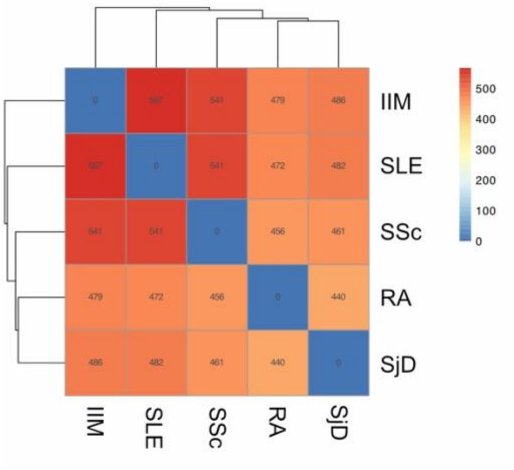

***Supplementary Fig. S2*:*Higher protein levels across diseases compared to healthy controls*.** *Heat map showing the number of overlapping proteins from each individual SARD that had higher levels (adjusted p-value < 0.01, logFC > 0.25) compared to the healthy control.*

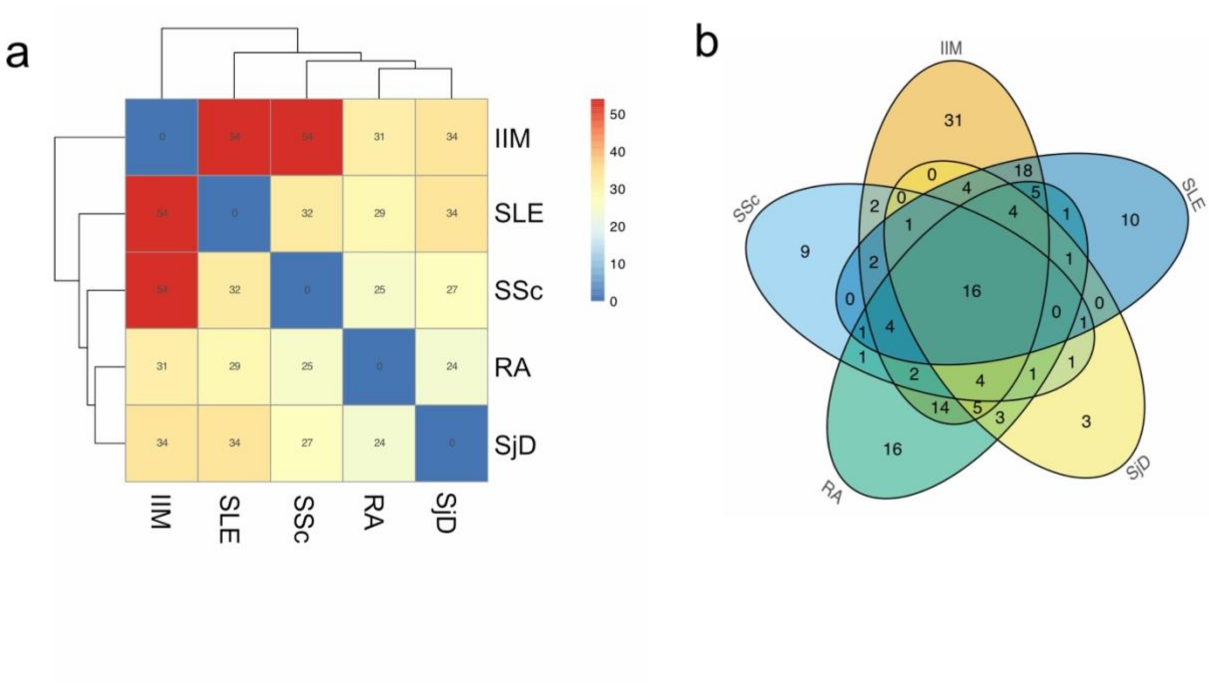

Supplementary Fig. S3*: *Lower proteins levels across diseases compared to healthy controls*.** ***a*) Heat map showing the number of overlapping proteins from each individual SARD that had lower levels (adjusted p-value < 0.01, logFC > -0.25) compared to the healthy control;* ***b****) Venn diagram showing the number of overlapping proteins from each individual SARD that had lower levels (adjusted p-value < 0.01, logFC > -0.25) compared to the healthy control.*

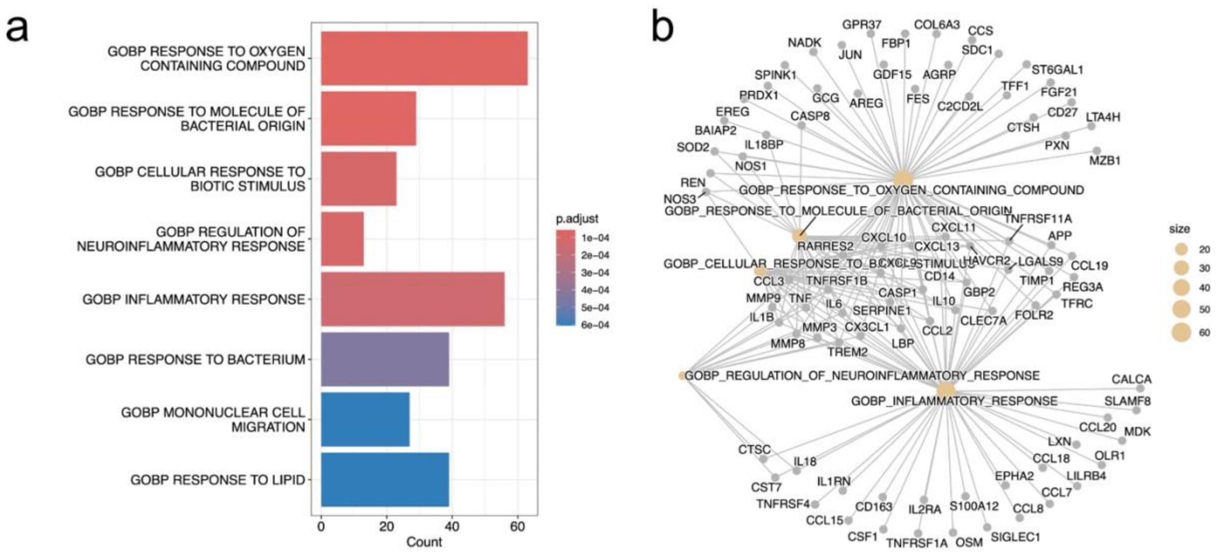

Supplementary Fig. S4: Grouped SARDs compared to healthy controls. Gene set enrichment of a*) barplot and* ***b****) gene-category network of proteins that had higher levels (adjusted p-value < 0.01, logFC > 0.25) in the grouped SARDs against the healthy control.*

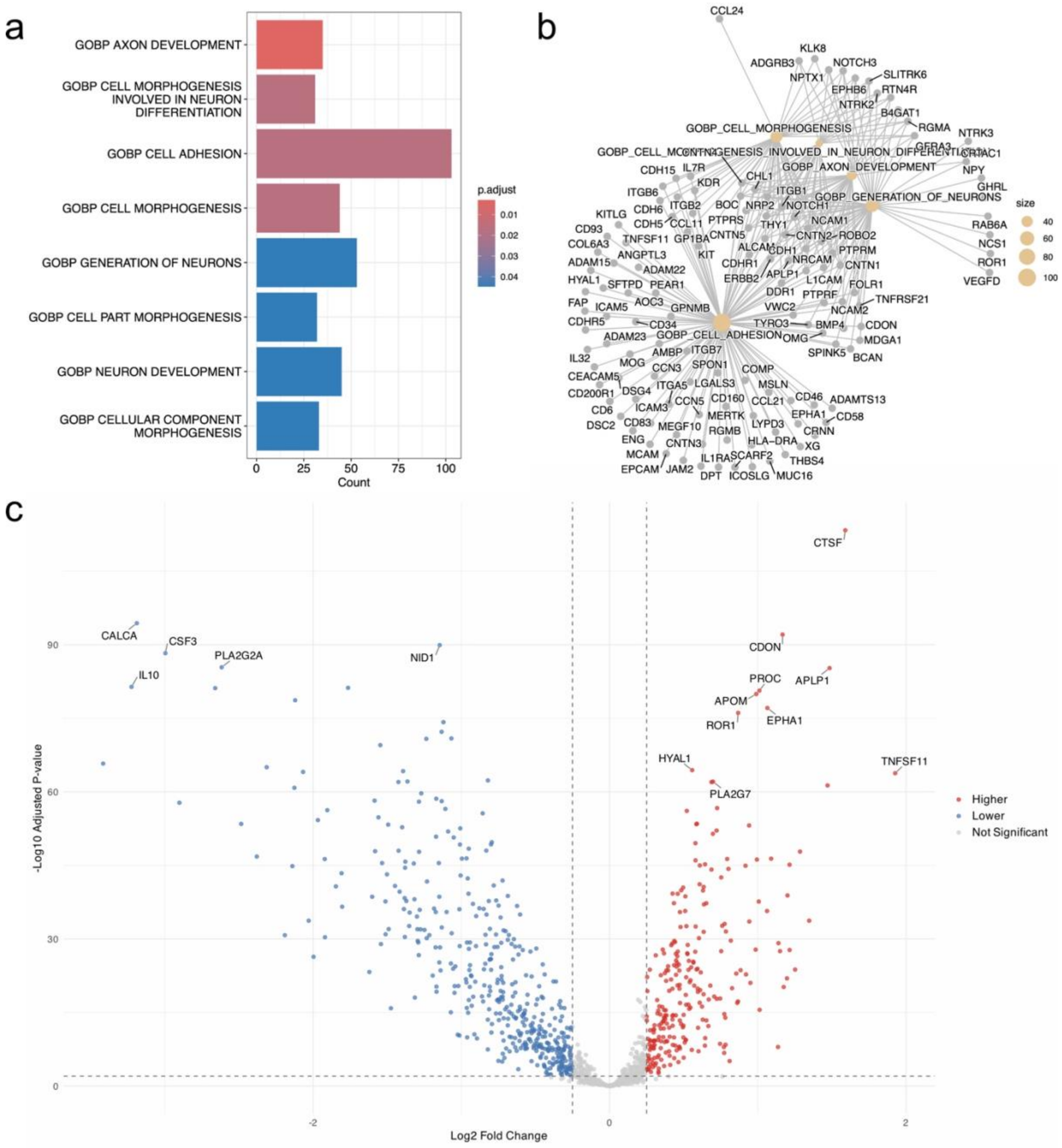

Supplementary Fig. S5*: *Grouped SARDs compared to infectious disease controls. *Gene set enrichment barplot from proteins that had higher levels (adjusted p-value < 0.01, logFC > 0.25) in the grouped SARDs than the infectious disease controls represented in **a**) barplot and **b**) gene-category network. **c**) Volcano plot highlighting proteins with differential levels in autoimmune cohort in comparison to grouped infectious disease. Proteins with significantly higher levels (adjusted p-value < 0.01, logFC > 0.25) in autoimmune cohort are shown in red, and lower levels (adjusted p-value < 0.01, logFC < -0.25) against infectious disease in blue.*

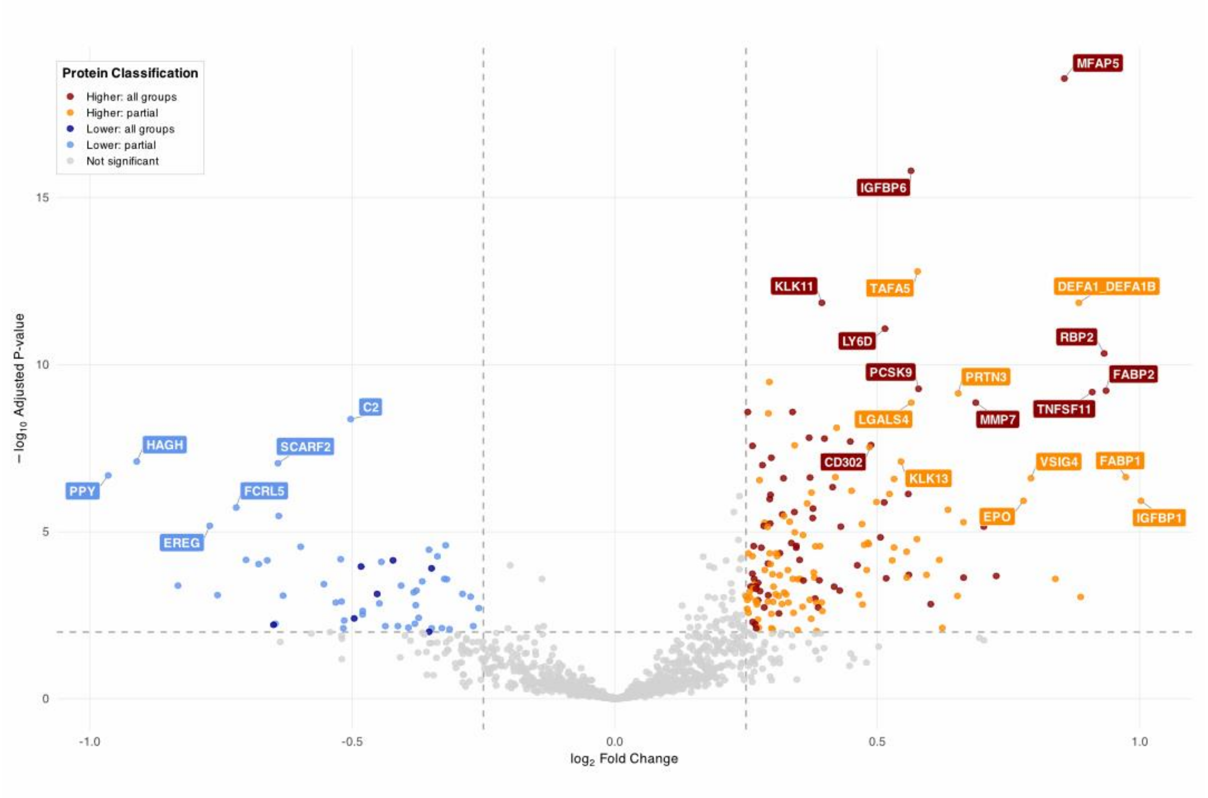

Supplementary Fig. S6: *Volcano plot for comparison of systemic lupus erythematosus (SLE) to the grouped autoimmune diseases (adjusted p-value < 0.01, logFC > 0.25), annotated by comparisons to healthy controls and grouped infectious disease controls. Dark red shows proteins significantly higher in SLE compared to all other groups (other autoimmune disease, healthy controls and infectious disease controls), yellow shows proteins significantly higher in SLE than autoimmune either alone or in combination with one other group (healthy controls or infectious disease control) Dark blue shows proteins significantly lower in SLE compared to all groups and light blue shows proteins significantly lower in SLE compared to autoimmune either alone or in combination with one other group (healthy controls or infectious disease control).*

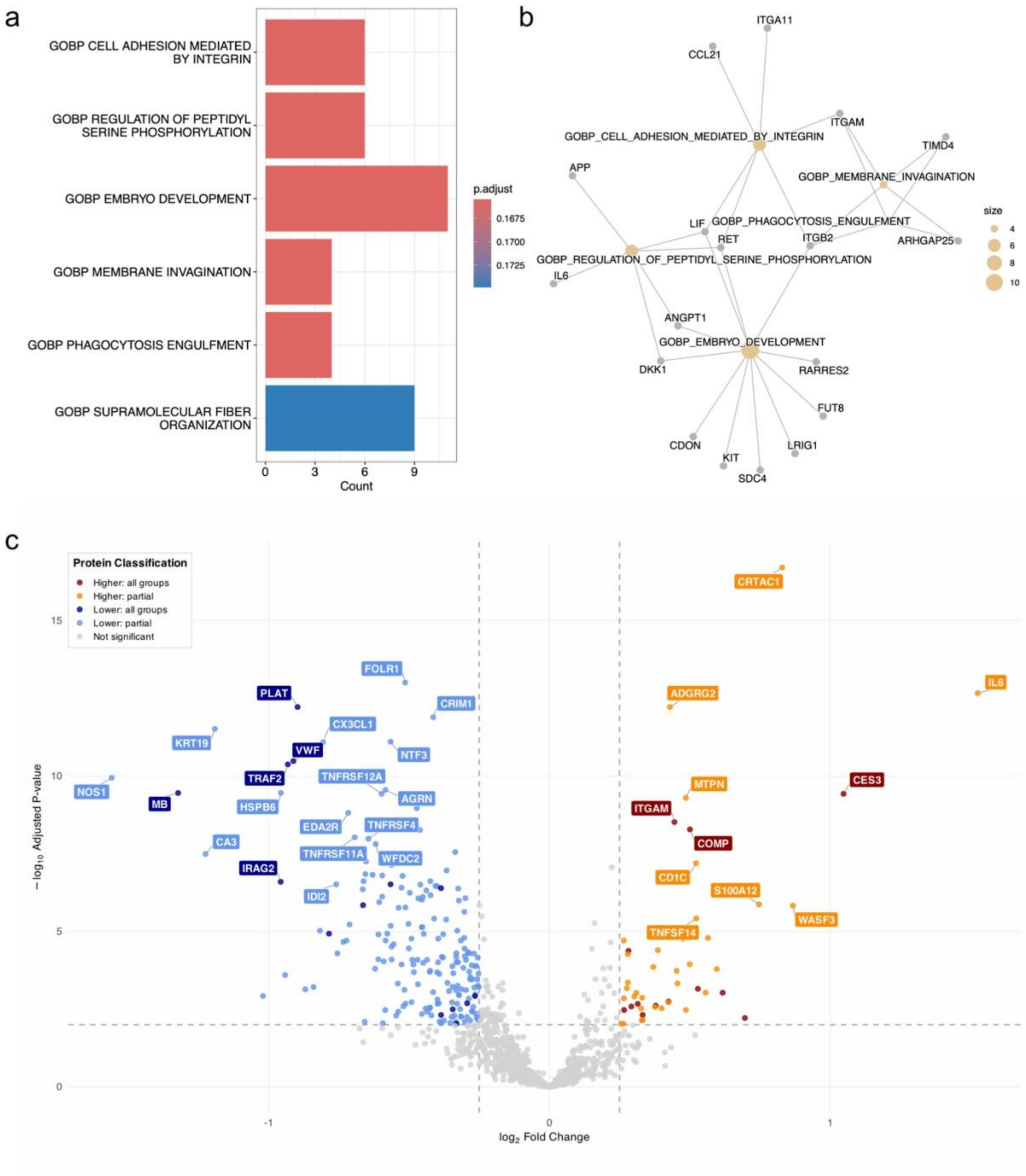

Supplementary Fig. S7: *Gene set enrichment of gene ontology biological processes from proteins that had higher levels (adjusted p-value < 0.01, logFC > 0.25) in rheumatoid arthritis (RA) compared to the grouped autoimmune diseases represented in* ***a****) barplot and* ***b****) gene-category network.* ***c****) Volcano plot for comparison of RA to the grouped autoimmune diseases (adjusted p-value <0.01, logFC > 0.25), annotated by comparisons to healthy controls and grouped infectious disease controls. Dark red shows proteins significantly higher in RA compared to all other groups (other autoimmune disease, healthy controls and infectious disease controls), yellow shows proteins significantly higher in RA than autoimmune either alone or in combination with one other group (healthy controls or infectious disease control). Dark blue shows proteins significantly lower in RA compared to*

all groups and light blue shows proteins significantly lower in RA compared to autoimmune either alone or in combination with one other group (healthy controls or infectious disease control).

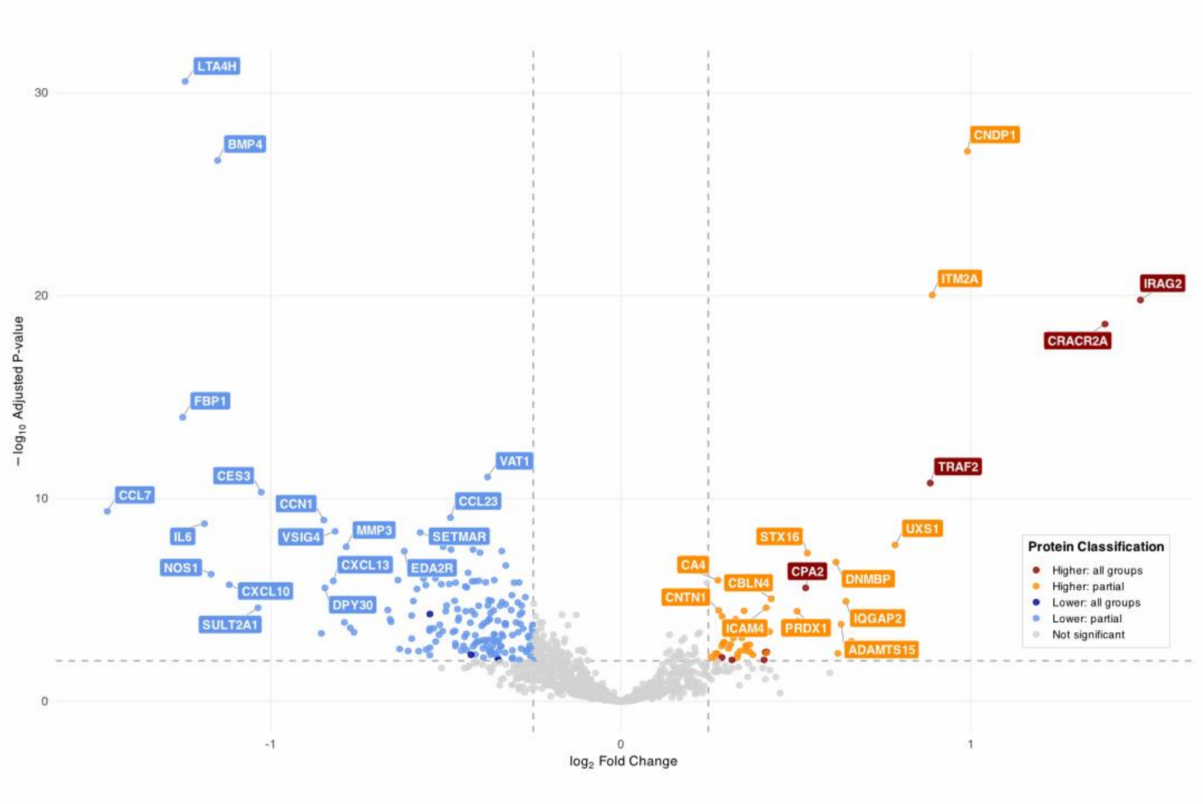

Supplementary Fig. S8: *Volcano plot for comparison of Sjögren disease (SjD) to the grouped autoimmune diseases (adjusted p-value <0.01, logFC > 0.25), annotated by comparisons to healthy control and grouped infectious disease controls. Dark red shows proteins significantly higher in SjD compared to all other groups (other autoimmune disease, healthy controls and infectious disease controls), yellow shows proteins significantly higher in SjD than autoimmune either alone or in combination with one other group (healthy controls or infectious disease control). Dark blue shows proteins significantly lower in SjD compared to all groups and light blue shows proteins significantly lower in SjD compared to autoimmune either alone or in combination with one other group (healthy controls or infectious disease control).*

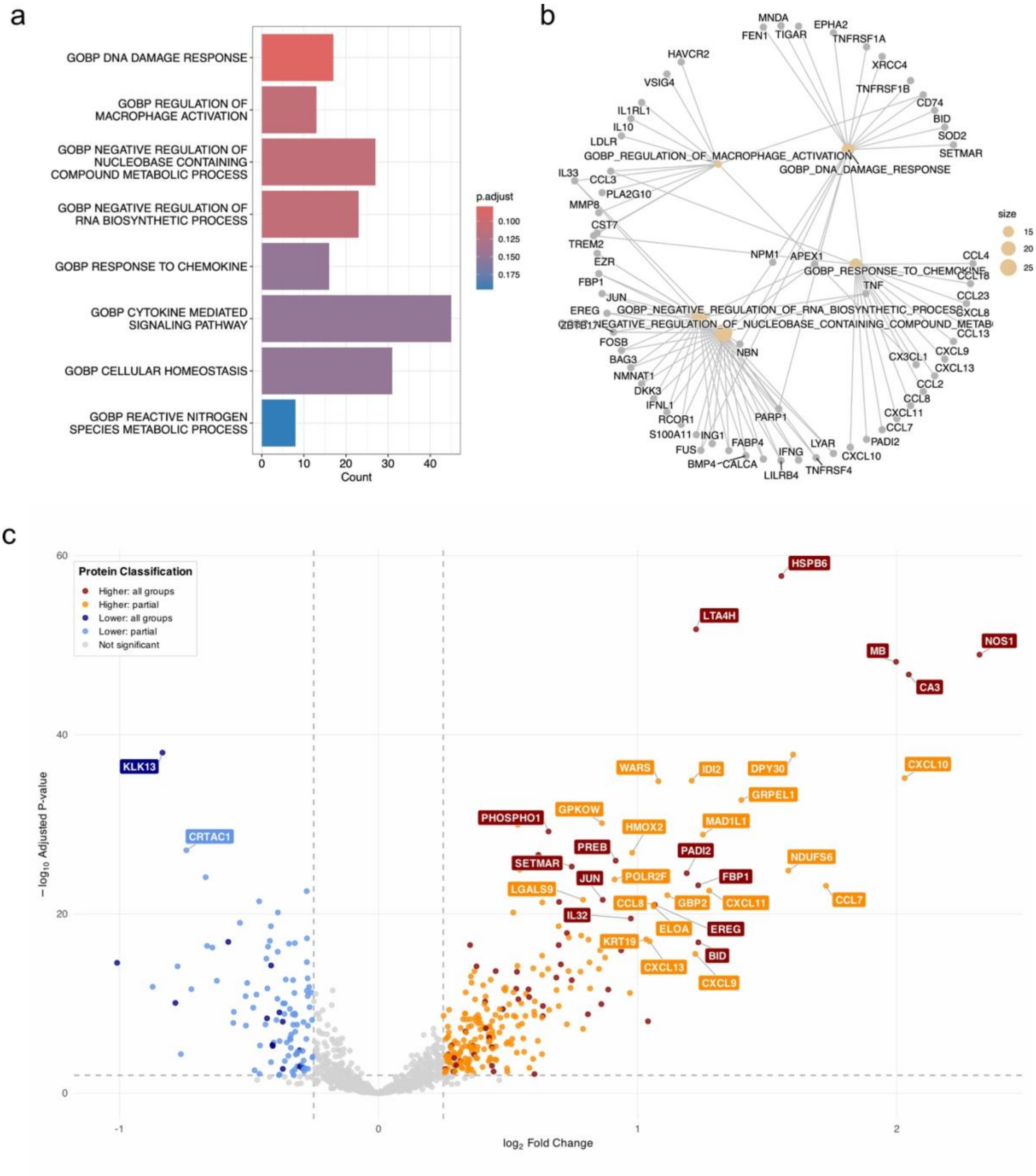

Supplementary Fig. S9: *Gene set enrichment of gene ontology biological processes from proteins that had higher levels (adjusted p-value < 0.01, logFC > 0.25) in idiopathic inflammatory myopathies (IIM) compared to the grouped autoimmune diseases represented in **a**) barplot and **b**) gene-category network. **c**) Volcano plot for comparison of IIM to the grouped autoimmune diseases (adjusted p-value <0.01, logFC > 0.25), annotated by comparisons to healthy controls and grouped infectious disease controls. Dark red shows proteins significantly higher in IIM compared to all other groups (other autoimmune disease, healthy controls and infectious disease controls), yellow shows proteins significantly higher in IIM than autoimmune either alone or in combination with one other group (healthy controls or infectious disease control). Dark blue shows proteins significantly lower in IIM*

compared to all groups and light blue shows proteins significantly lower in IIM compared to autoimmune either alone or in combination with one other group (healthy controls or infectious disease control).

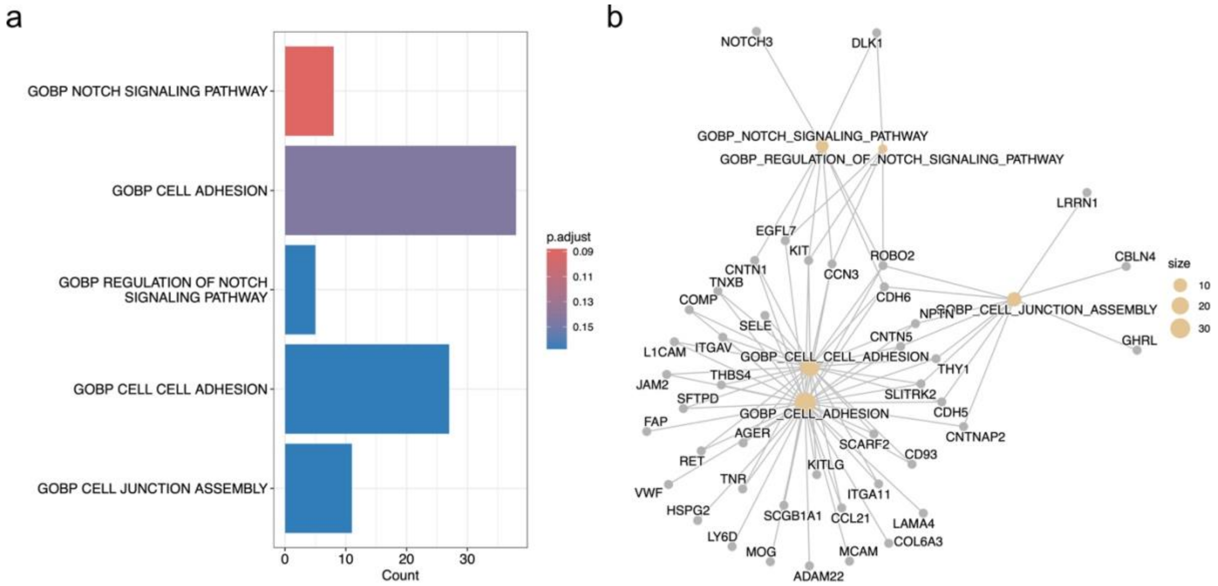

Supplementary Fig. S10: *Gene set enrichment of gene ontology biological processes from proteins that had higher levels (adjusted p-value < 0.01, logFC > 0.25) in systemic sclerosis (SSc) compared to the grouped autoimmune diseases represented in* ***a****) barplot and* ***b****) gene-category network.*
