# Supplementary material for: Pan-disease blood protein profiles of rheumatic autoimmune diseases
Source: Commun Med (Lond). 2026 Jul 13;6:390. doi: 10.1038/s43856-026-01779-0 (PMC13365237; doi:10.1038/s43856-026-01779-0)
Supplement: Supplementary file 2 — Description of Additional Supplementary files [file 43856_2026_1779_MOESM2_ESM.docx]

**Description of Additional Supplementary Files**

Supplementary Data 1: differential analysis results

Supplementary Data 2: machine learning results

Supplementary Data 3: overlap results

Supplementary Data 4: source data for figures
